# Risk detection and risk management for diabetes patients with atrial fibrilation

**DOI:** 10.1186/1878-5085-5-S1-A71

**Published:** 2014-02-11

**Authors:** Matthias M Weber, Stephan von Bandemer

**Affiliations:** 1 Endocrinology and metabolic diseases, University Medicine Mainz, Johannes Gutenberg-Universität, Langenbeckstraße 1, 55131 Mainz, Germany; 2 Institut Arbeit und Technik, Munscheidstr. 14, 45886 Gelsenkirchen, Germany

## Scientific objectives

Diabetes is considered as a risk factor for atrial fibrillation and patients with a co morbidity of diabetes and atrial fibrillation have an increased risk of cardio-vascular complications like stroke. However, paroxysmal atrial fibrillation often is not recognized and therefore stroke is a frequent and severe complication of diabetes and atrial fibrillation. The objective of the study is a screening of patients with diabetes mellitus type 2 for atrial fibrillation and as a consequence an individually adjusted risk management regarding glucoses levels, anticoagulation and blood pressure.

## Technical approaches

To detect atrial fibrillation ECGs of diabetes patients will be evaluated by an automated AF episode detection algorithm that already has proven superiority in the detection of atrial fibrillation. The software uses an algorithm that detects QRS complexes of the ECG data and then classifies them as being of atrial or ventricular origin and finally creates a list of R-R-intervals. To detect episodes of AF, the software performs a time series analysis of multiple mathematical parameters that are typical for an absolute arrhythmia during AF. Based on this analysis, the system creates a report on whether episodes of AF are present [2]. The procedure is suggested to be applied in a multi-center study and will be used as a basis for the design and evaluation of an individually risk adjusted medication of patients. This will especially combine glucose level and blood pressure control and anticoagulation strategies.

## Expected results

The study is expected to give detailed information on the increased prevalence of atrial fibrillation in patients with type 2 diabetes and provides the data base for an optimized therapy regime in order to avoid cardio-vascular complications.

## Outlook and recommendations

The detection of atrial fibilation as a major risk factor for cardio-vascular complications of diabetic patients will have to be improved. This strategy can be combined with other strategies of detection of complication risks (especially proteomics) and improve personalized treatment of diabetic patients.
